# Association between triglyceride to high-density lipoprotein cholesterol ratio and nonalcoholic fatty liver disease and liver fibrosis in American adults: an observational study from the National Health and Nutrition Examination Survey 2017–2020

**DOI:** 10.3389/fendo.2024.1362396

**Published:** 2024-07-16

**Authors:** Jianjun Wang, Han Li, Xiaoyi Wang, Ruizi Shi, Junchao Hu, Xintao Zeng, Hua Luo, Pei Yang, Huiwen Luo, Yuan Cao, Xianfu Cai, Sirui Chen, Decai Wang

**Affiliations:** ^1^ Department of Hepatobiliary Surgery, Mianyang Central Hospital, School of Medicine, University of Electronic Science and Technology of China, Mianyang, China; ^2^ NHC Key Laboratory of Nuclear Technology Medical Transformation, Mianyang Central Hospital, School of Medicine, University of Electronic Science and Technology of China, Mianyang, China; ^3^ Department of Cardiology, The Fifth Hospital of Wuhan, Wuhan, China; ^4^ Department of Neurosurgery, Mianyang Central Hospital, School of Medicine, University of Electronic Science and Technology of China, Mianyang, China; ^5^ Department of Urology, Mianyang Central Hospital, School of Medicine, University of Electronic Science and Technology of China, Mianyang, China

**Keywords:** National health and nutrition examination survey, nonalcoholic fatty liver disease, cross-sectional study, triglyceride to high-density lipoprotein cholesterol ratio, liver fibrosis

## Abstract

**Objective:**

This study investigated the link between triglyceride to high-density lipoprotein cholesterol (TG/HDL-C) ratio and nonalcoholic fatty liver disease (NAFLD) and liver fibrosis in American adults.

**Methods:**

Information for 6495 participants from the National Health and Nutrition Examination Survey (NHANES) 2017–2020.03 was used for this cross-sectional study. The link between TG/HDL-C ratios and NAFLD and liver fibrosis was assessed by multiple linear regression before evaluating nonlinear correlations based on smoothed curve fitting models. Stratification analysis was then applied to confirm whether the dependent and independent variables displayed a stable association across populations.

**Results:**

TG/HDL-C ratios were positively correlated with NAFLD, with higher ratios being linked to increased prevalence of NAFLD. After adjusting for potential confounders, the odds ratios (OR) for NAFLD patients in the fourth TG/HDL-C quartile were 3.61 (95% confidence interval [CI], 2.94–4.38) (*P* for trend < 0.001) in comparison with those in the first quartile after adjusting for clinical variables. However, no statistical significance was noted for the ratio for liver fibrosis after adjusting for potential confounders (*P* for trend = 0.07). A nonlinear correlation between TG/HDL-C ratios and NAFLD was observed based on smoothed curve fitting models. However, a nonlinear relationship between the ratios and liver fibrosis was not established. In subgroup analyses, there was an interaction between smoking status and TG/HDL-C ratio in relation to the prevalence of liver fibrosis (*P* for interaction < 0.001).

**Conclusions:**

Among American adults, the TG/HDL-C ratio was noted to be nonlinearly positively associated with the prevalence of NAFLD; however, this relationship was not present in liver fibrosis.

## 1 Introduction

Nonalcoholic fatty liver disease (NAFLD), as a globally prevalent metabolic-related disease of the liver, affects approximately 38% of the world’s population, and as such, it significantly threatens public health (1). Most patients with NAFLD do not experience discomfort, whereas a small percentage may present with fatigue, epigastric discomfort, or vague pain (2). While its typical characteristic include excessive lipid accumulation in the liver without alcohol abuse, some NAFLD patients can further progress to nonalcoholic steatohepatitis, liver fibrosis, cirrhosis, and eventually hepatocellular carcinoma. The number of individuals at risk for this serious consequence is steadily increasing (3, 4). Although NAFLD involves complex risk factors, its prevalence is usually associated with obesity (5–7). NAFLD is associated with poor hepatic prognosis and an increased risk of extrahepatic metabolic abnormalities, including hyperlipidemia, hyperglycemia, insulin resistance (IR), and hyperuricemia (8, 9). Furthermore, cardiovascular events and extrahepatic tumorigenesis, the leading causes of extrahepatic death, are also more likely with this condition (8, 10). Despite ongoing efforts to determine drugs to treat NAFLD and liver fibrosis, there are no specific licensed drugs that can completely reverse NAFLD or liver fibrosis. The main goals of current treatment are to ameliorate metabolic sequelae and to control the risk of cardiovascular events to reduce associated mortality (11, 12). Therefore, it is of utmost importance to investigate novel treatment agents, targets, and interventional approaches for NAFLD and liver fibrosis.

The severity of hepatic steatosis and liver fibrosis is currently assessed by liver biopsy, but despite being an established standard, this practice is invasive and expensive, with limited acceptability, and hence, it is not only impractical for widespread screening of the general population but also requires caution in clinical practice (13). Therefore, there is an urgent need to identify new noninvasive tests to diagnose NAFLD. Serum markers and their combinations have several advantages. They are easy to access, low-cost, and offer high diagnostic accuracy, making them valuable for disease screening. A combination of lipids and lipoproteins proves to be more beneficial for predicting risks of NAFLD compared with individual lipid values as it captures interactions between lipid components (14, 15). Patients with NAFLD usually have lipid metabolism disorders and dyslipidemia, and serum markers may show reduced levels of high-density lipoprotein cholesterol (HDL-C) along with increased levels of low-density lipoprotein cholesterol (LDL-C), total cholesterol (TC), and triglycerides (TG) (16). TG level is crucial for NAFLD development and progression. When TG level is elevated in the blood, there is an increase in the amount of TG celiac “debris” and free fatty acids transported to the liver through the bloodstream, which exceeds the liver’s ability to utilize these fatty acids, resulting in fatty infiltration of hepatocytes (17). In hepatocytes from patients with NAFLD, intracellular TG levels exceed 5% (18). As free fatty acids are oxidized in the mitochondria, hepatocytes experience oxidative stress. The excess reactive oxygen species generated in these reactions harm the hepatocytes, impairing their ability to metabolize free fatty acids and aggravating oxidative stress and lipid peroxidation, ultimately hastening the pathological process of NAFLD (19). The development of NAFLD impairs the ability of hepatocytes to oxidize, transport, and resynthesize lipids in the blood, exacerbating the increase in TG levels (20). HDL-C is essential for preventing atherosclerosis through the reverse cholesterol transport system that carries dietary cholesterol from peripheral tissues to the liver, where it is transformed into bile salts and expelled in the feces (21). HDL-C even possesses antioxidant and anti-inflammatory properties based on which it is called “good cholesterol.” Additionally, HDL-C levels showed a negatively correlated with NAFLD diagnosis (22).

The TG/HDL-C ratio exhibits a strong association with IR (23, 24). Indeed, in a study involving a multiethnic primary prevention cohort, a stronger relationship was noted between TG/HDL-C and a homeostasis model assessment of IR in comparison with other lipid parameters (25). Moreover, it can predict cardiovascular diseases, hypertension, and type 2 diabetes (26–28). Limited evidence has recently suggested that this ratio could also be linked to NAFLD, with an observational study noting an increased likelihood of developing NAFLD in children and adolescents having higher TG/HDL-C ratios (29). Similarly, in Japan, a population-based cohort study demonstrated that TG/HDL-C ratios could predict NAFLD incidence (30). Nonetheless, the relationship between the rate and NAFLD and liver fibrosis in American adults remains unclear.

To the best of the current authors’ knowledge, no epidemiological studies have explored the above association in American adults. Therefore, based on information of 6495 participants from the National Health and Nutrition Examination Survey (NHANES) 2017–2020.03, a cross-sectional study was performed for investigating how TG/HDL-C ratio was linked to NAFLD and liver fibrosis. It is expected that this study will provide new understanding of NAFLD that will guide treatment and management of the condition.

## 2 Materials and methods

### 2.1 Data sources

Data from the 2017–2020.03 NHANES database, a comprehensive population-based survey, was used for the current observational study. Information on the health, diet as well as socioeconomic, and demographic characteristics of the participants, were obtained through questionnaires, physical examinations, laboratory tests, and medical examinations, including anthropometric measurements and laboratory evaluations. The National Center for Health Statistics Ethics Review Committee gave approval for the NHANES survey protocol, with written informed consent also obtained from all participants. Since the NHANES database is publicly available, this study was exempted from ethical review.

### 2.2 Inclusion/exclusion criteria for participant selection

**Figure 1:**
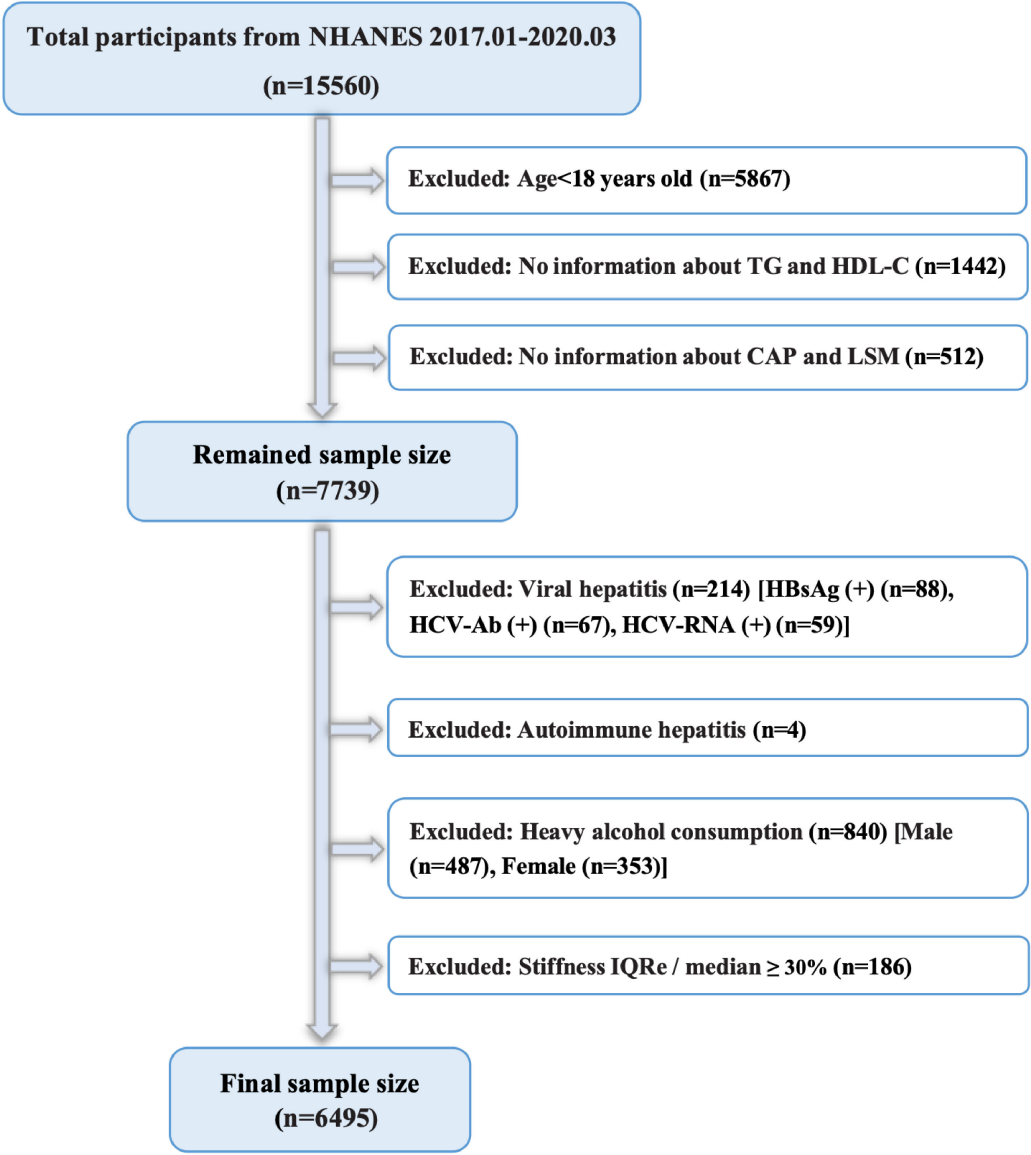
Flowchart for the participants.

Out of 15,560 American adults included from the NHANES database, participants below 18 years old (n = 5867) who did not undergo the liver ultrasound transient elastography (LUTE) test (n = 512) and with missing TG/HDL-C ratio information (n = 1442) were then excluded alongside those who had a history of autoimmune hepatitis (n = 4) or viral hepatitis (n = 214), with the latter including individuals who were positive for hepatitis C virus RNA (n = 59), hepatitis C antibody (n = 67), and hepatitis B surface antigen (n = 88). In addition, males and females with an alcohol intake of more than 30 g/d (n = 487) and 20 g/d (n = 353), respectively, indicative of substantial alcohol consumption, were not considered (31). Finally, according to the NHANES guidelines, individuals with liver stiffness measurements (LSMs) displaying an interquartile range/median > 30% were deemed unreliable and were, therefore, excluded (n = 186). As a result, this study involved 6495 participants (**Figure 1**).

### 2.3 Demographic and laboratory variables

Demographic characteristics, such as age, sex, ethnicity, educational attainment, smoking and drinking status, body mass index (BMI), household income, family income to poverty threshold ratio as well as past medical history of blood transfusion, hypertension, and diabetes, were retrieved from the NHANES database.

Laboratory variables included TG, TC, albumin (Alb), glycated hemoglobin (HbA1c), aspartate aminotransferase (AST), γ-glutamyltransferase (GGT), C-reactive protein (CRP), alanine aminotransferase (ALT), LDL-C, HDL-C, and ferritin. The measurement protocols for these variables were obtained from the literature (32, 33). The ratio of TG to HDL-C was used to obtain the TG/HDL-C ratio.

### 2.4 Evaluation of NAFLD and liver fibrosis

LUTE primarily aims to provide an objective measure of liver steatosis (liver fat) and liver fibrosis (liver scarring) which are two critical manifestations of liver disease. A FibroScan^®^ machine was used for all measurements, where the assessment of one physical parameter, referred to as the controlled attenuation parameter (CAP), primarily reflected the extent of hepatic steatosis. Steatosis was considered in cases where the median CAP value was ≥ 274 dB/m (34). Liver fibrosis was determined based on LSMs and most recent guidelines of the European Association for the Study of the Liver (35). Based on thresholds of 8.2, 9.7, and 13.6 kPa, liver fibrosis was eventually categorized as F2, F3, and F4, respectively (32, 36).

### 2.5 Statistical analyses

In March 2020, field operations for the NHANES project were halted because of the 2019 coronavirus pandemic. Therefore, to construct a nationally representative sample, information obtained from 2019 to March 2020 and that from the NHANES 2017–2018 cycle was combined. Additionally, for the pre-epidemic data files from March 2017 to 2020, a special weighting procedure was employed by the NHANES workgroup. LUTE data were analyzed using NHANES check sample weights in accordance with the NHANES guidelines, with special examination sample weights (variable name: WTMECPRP) employed for the 2017–2020.03 cycle. R software (version 4.2.2) was used to extract, merge, clean, and analyze the NHANES dataset, with *P*-values < 0.05 indicating statistical significance. Categorizing the TG/HDL-C ratios into quartiles yielded four participant subgroups. For normally distributed continuous variables, data are presented as mean ± standard deviation (SD); for non-normally distributed continuous variables, data are presented as median (interquartile range, IQR); and for categorical variables, data are presented as numbers (%). Furthermore, differences between categorical data of the four subgroups were compared with chi-squared tests, while one-way analysis of variance and Kruskal–Wallis were used in the case of normally and non-normally distributed data, respectively. Correlations between TG/HDL-C ratio and clinical variables were then determined with Spearman’s correlation analyses before investigating the link between the independent and dependent variables based on multiple linear regression analysis which generated three models with varying covariates adjustments: For Model 1, no adjustments were made for covariates; Adjustments for age, sex, and ethnicity were made in the case of Model 2; and Model 3, additionally adjusted for smoking status, poverty income ratio (PIR), educational attainment, diabetes, hypertension, BMI, HbA1c and TC levels. To evaluate the stability of the associations across cohorts and to identify sensitive populations, we conducted subgroup analyses with subgroups that included age, gender, ethnicity, BMI, educational level, household income, smoking status, and diabetes. Finally, the existence of a nonlinear association between the independent and dependent variables was assessed by smoothed curve fitting analysis.

## 3 Results

### 3.1 Participants’ characteristics

In total, 6495 participants were included in this study (**Table 1**). Eventually, this study included 3108 males and 3387 females. Hepatic fibrosis and NAFLD were observed in 9.3% and 42.5% of the participants, respectively. Higher TG/HDL-C ratios were linked to a higher prevalence of NAFLD, severe hepatic steatosis, and hepatic fibrosis (*P* < 0.05). Age, sex, ethnicity, smoking status, household income, educational attainment, BMI as well as diabetes, hypertension, TC, LDL-C, ALT, AST, GGT, Alb, HbA1c, CRP, and ferritin levels were significant factors in all TG/HDL-C quartiles (*P* < 0.05). However, the TG/DHL-C quartiles were not significantly different for blood transfusion (*P* = 0.09).

**Table 1:** Clinical characteristics for the study participants.

Variable	Total (n=6495)	Q1 (n=1623)	Q2 (n=1625)	Q3 (n=1624)	Q4 (n=1623)	*P* value
TG/HDL-C		0.13-0.59	0.59-0.95	0.95-1.59	1.59-28.52	
Age, years	50 (33-64)	43 (28-61)	51 (33-64)	54 (37-66)	51 (38-63)	<0.001
Male, n (%)	3108 (47.8)	616 (38.0)	672 (41.4)	804 (49.5)	1016 (62.6)	<0.001
BMI, kg/m^2^	28.5 (24.7-33.5)	25.1 (21.9-29.4)	28.1 (24.2-33.1)	29.8 (26.2-34.8)	30.9 (27.0-35.4)	<0.001
Ethnicity, n (%)						<0.001
Non-Hispanic White	832 (12.8)	128 (7.9)	192 (11.8)	235 (14.5)	277 (17.1)	
Non-Hispanic Black	1856 (28.6)	411 (25.3)	415 (25.5)	478 (29.4)	552 (34.0)	
Mexican American	2185 (33.6)	465 (28.6)	525 (32.3)	577 (35.5)	618 (38.1)	
Other Race	1622 (25.0)	619 (38.1)	493 (30.3)	334 (20.6)	176 (10.8)	
Education, n (%)						<0.001
Less than high school	1517 (23.4)	335 (20.6)	349 (21.5)	406 (25.0)	427 (26.3)	
High school	1485 (22.9)	349 (21.5)	378 (23.2)	379 (23.3)	379 (23.4)	
More than high school	3493 (53.8)	939 (57.9)	898 (55.3)	839 (51.7)	817 (50.3)	
Household income, n (%)						0.02
PIR<1	1115 (17.2)	284 (17.5)	282 (17.4)	271 (16.7)	278 (17.1)	
PIR 1 to <3	2370 (36.5)	558 (34.4)	613 (37.7)	590 (36.3)	609 (37.5)	
PIR ≥3	2093 (32.2)	558 (34.4)	532 (32.7)	494 (30.4)	509 (31.4)	
Unclear	917 (14.1)	223 (13.7)	198 (12.2)	269 (16.6)	227 (14.0)	
Smoking status, n (%)						<0.001
Former smoker	1429 (22.0)	290 (17.9)	322 (19.8)	390 (24.0)	427 (26.3)	
Current smoker	1008 (15.5)	221 (13.6)	242 (14.9)	256 (15.8)	289 (17.8)	
Never smoker	4058 (62.5)	1112 (68.5)	1061 (65.3)	978 (60.2)	907 (55.9)	
Diabetes, n (%)	953 (14.7)	110 (6.8)	186 (11.4)	284 (17.5)	373 (23.0)	<0.001
Hypertension, n (%)	2262 (34.8)	425 (26.2)	538 (33.1)	626 (38.6)	673 (41.5)	<0.001
Blood transfusion, n (%)	658 (10.1)	147 (9.1)	151 (9.3)	179 (11.0)	181 (11.2)	0.09
TG, mmol/L	1.3 (0.9-1.8)	0.7 (0.6-0.8)	1.0 (0.9-1.2)	1.5 (1.3-1.7)	2.5 (2.0-3.2)	<0.001
TC, mmol/L	4.7 (4.0-5.4)	4.5 (3.9-5.6)	4.6 (4.0-5.3)	4.7 (4.0-5.4)	5.0 (4.3-5.7)	<0.001
HDL-C, mmol/L	1.3 (1.1-1.6)	1.7 (1.5-1.9)	1.4 (1.2-1.6)	1.2 (1.1-1.4)	1.0 (0.9-1.1)	<0.001
LDL-C, mmol/L	2.8 (2.2-3.4)	2.5 (2.0-3.0)	2.8 (2.2-3.4)	3.0 (2.4-3.6)	3.0 (2.4-3.6)	<0.001
ALT, U/L	17.0 (13.0-25.0)	14.0 (11.0-20.0)	16.0 (12.0-22.0)	18.0 (13.0-26.0)	22.0 (16.0-32.0)	<0.001
AST, U/L	19.0 (16.0-23.0)	18.0 (15.0-22.0)	18.0 (15.0-22.0)	19.0 (16.0-23.0)	20.0 (16.0-32.0)	<0.001
GGT, U/L	20.0 (14.0-30.0)	16.0 (12.0-23.0)	18.0 (13.0-27.0)	21.0 (15.0-31.0)	26.0 (19.0-41.0)	<0.001
Alb, g/L	41.0 (39.0-43.0)	41.0 (39.0-43.0)	41.0 (39.0-43.0)	41.0 (38.0-43.0)	41.0 (39.0-43.0)	0.001
HbA1c, %	5.6 (5.3-6.0)	5.4 (5.2-5.7)	5.5 (5.3-5.9)	5.7 (5.4-6.1)	5.7 (5.4-6.4)	<0.001
CRP, mg/L	1.9 (0.8-4.4)	1.0 (0.5-2.7)	1.9 (0.8-4.5)	2.3 (1.0-5.2)	2.5 (1.2-5.2)	<0.001
Ferritin, ng/mL	103.0 (49.0-192.0)	78.7 (35.6-141.0)	96.7 (44.6-177.5)	114.0 (55.8-208.0)	136.0 (69.7-241.0)	<0.001
CAP, dB/m	261.0 (216.0-307.0)	219.0 (192.0-256.0)	248.0 (211.0-288.0)	277.0 (235.0-316.0)	301.0 (259.0-341.0)	<0.001
LSM, kPa	5.0 (4.1-6.1)	4.6 (3.9-5.6)	4.8 (4.0-5.9)	5.0 (4.1-6.3)	5.5 (4.4-6.6)	<0.001
NAFLD, n (%)	2762 (42.5)	296 (18.2)	526 (32.4)	857 (52.8)	1083 (66.7)	<0.001
Severe hepatic steatosis, n (%)	1788 (27.5)	137 (8.4)	296 (18.2)	551 (33.9)	804 (49.5)	<0.001
Liver fibrosis, n (%)	602 (9.3)	79 (4.9)	110 (6.8)	193 (11.9)	220 (13.6)	<0.001
F2, n (%)	234 (3.6)	39 (2.4)	42 (2.6)	70 (4.3)	83 (5.1)	
F3, n (%)	210 (3.2)	21 (1.3)	37 (2.3)	69 (4.2)	83 (5.1)	
F4, n (%)	158 (2.4)	19 (1.2)	31 (1.9)	54 (3.3)	54 (3.3)	

Normally distributed values in the table are given as the mean ± SD, skewed distributed values are given as the median (25 and 75% interquartiles), and categorical variables are given as frequency (percentage).

TG/HDL-C, triglyceride to high-density lipoprotein cholesterol ratio; BMI, body mass index; PIR, poverty income ratio; TC, total cholesterol; TG, triglyceride; HDL-C, high-density lipoprotein cholesterol; LDL-C, low-density lipoprotein cholesterol; ALT, alanine aminotransferase; AST, aspartate aminotransferase; GGT, γ-glutamyltransferase; Alb, albumin; HbA1c, glycated hemoglobin; CRP, C-reactive protein; CAP, controlled attenuation parameter; LSM, liver stiffness measurements; NAFLD, nonalcoholic fatty liver disease.

### 3.2 Associations between TG/HDL-C ratios and clinical variables

A positive correlation was noted between TG/DHL-C ratio and age, BMI, LSM, CAP, HbA1c, GGT,TC, LDL-C, ALT, AST, ferritin levels; and (*r* = 0.041, 0.156, 0.079, 0.309, 0.211, 0.151, 0.187, 0.113, 0.199, 0.091, 0.144, and respectively; *P* < 0.05) (**Table 2**). However, a significantly positive association was not observed with Alb and CRP levels (*r* = 0.023 and 0.022, and *P* = 0.06 and 0.08, respectively).

**Table 2:** Relationships between TG/HDL-C and clinical parameters.

Variable	r	*P* value
Age	0.041	0.001
BMI	0.156	<0.001
TC	0.187	<0.001
LDL-C	0.113	<0.001
ALT	0.199	<0.001
AST	0.091	<0.001
GGT	0.151	<0.001
Alb	0.023	0.06
HbA1c	0.211	<0.001
CRP	0.022	0.08
Ferritin	0.144	<0.001
CAP	0.309	<0.001
LSM	0.079	<0.001

r Spearman’s correlation coefficient.

TG/HDL-C, triglyceride to high-density lipoprotein cholesterol ratio; BMI, body mass index; TC, total cholesterol; TG, triglyceride; LDL-C, low-density lipoprotein cholesterol; ALT, alanine aminotransferase; AST, aspartate aminotransferase; GGT, γ-glutamyltransferase; Alb, albumin; HbA1c, glycated hemoglobin; CRP, C-reactive protein; CAP, controlled attenuation parameter; LSM, liver stiffness measurement.

### 3.3 Association between TG/HDL-C and NAFLD and liver fibrosis

All models indicated that TG/HDL-C ratios and NAFLD were positively correlated (**Table 3**). Grouping the TG/HDL-C ratios into quartiles revealed a consistent correlation, with the association between the two strengthening as the ratio increased (*P* for trend < 0.001). Participants in Q2, Q3, and Q4 exhibited NAFLD odds ratios (ORs) of 2.15 (95% confidence interval [CI], 1.82–2.53), 5.01 (95% CI, 4.27–5.87), and 8.99 (95% CI, 7.64–10.58), respectively, compared with those in Q1 (all *P* < 0.05). Moreover, following adjustments for additional clinical variables, the corresponding NAFLD ORs for Q2, Q3, and Q4 were 1.32 (95% CI, 1.09–1.59), 2.41 (95% CI, 2.00–2.90), and 3.61 (95% CI, 2.94–4.38), respectively, in comparison with the TG/HDL-C ratio of the Q1 participants (all *P* < 0.05).

**Table 3:** Association of TG/HDL-C with NAFLD and liver fibrosis.

Exposure	Model 1, OR (95% CI)	Model 2, OR (95% CI)	Model 3, OR (95% CI)
NAFLD			
TG/HDL-C	1.92 (1.81-2.04)	1.84 (1.73-1.96)	1.38 (1.30-1.47)
Quintiles of TG/HDL-C			
Q1	Reference	Reference	Reference
Q2	2.15 (1.82-2.53)	1.99 (1.69-2.39)	1.32 (1.09-1.59)
Q3	5.01 (4.27-5.87)	4.51 (3.82-5.31)	2.41 (2.00-2.90)
Q4	8.99 (7.64-10.58)	8.19 (6.91-9.71)	3.61 (2.94-4.38)
*P* for trend	<0.001	<0.001	<0.001
Liver fibrosis			
TG/HDL-C	1.16 (1.11-1.21)	1.15 (1.10-1.20)	1.06 (1.01-1.11)
Quintiles of TG/HDL-C			
Q1	Reference	Reference	Reference
Q2	1.42 (1.05-1.91)	1.32 (0.98-1.79)	0.89 (0.64-1.22)
Q3	2.64 (2.01-3.46)	2.41 (1.83-3.18)	1.18 (0.87-1.60)
Q4	3.06 (2.35-4.00)	2.87 (2.17-3.79)	1.20 (0.88-1.64)
*P* for trend	<0.001	<0.001	0.07

Model 1: unadjusted model.

Model 2: adjusted for age, sex and ethnicity.

Model 3: additionally adjusted for education, poverty income ratio, smoking status, diabetes, hypertension, BMI, HbA1c, and TC.

Additionally, TG/HDL-C ratio was positively correlated with liver fibrosis in Models 1 and 2 and was statistically significant, but this positive correlation was not statistically significant in Model 3 (**Table 3**). Q2, Q3, and Q4 participants exhibited ORs of 1.42 (95% CI, 1.05–1.91), 2.64 (95% CI, 2.01–3.46), and 3.06 (95% CI, 2.35–4.00) for liver fibrosis, respectively compared with the TG/HDL-C ratio of Q1 participants (*P* for trend < 0.001). However, following adjustments for other clinical variables in Model 3, the OR of liver fibrosis did not reach statistical significance despite enhanced association between TG/HDL-C and liver fibrosis at higher ratios (*P* for trend = 0.07).

### 3.4 Nonlinear relationship detection

**Figure 2:**
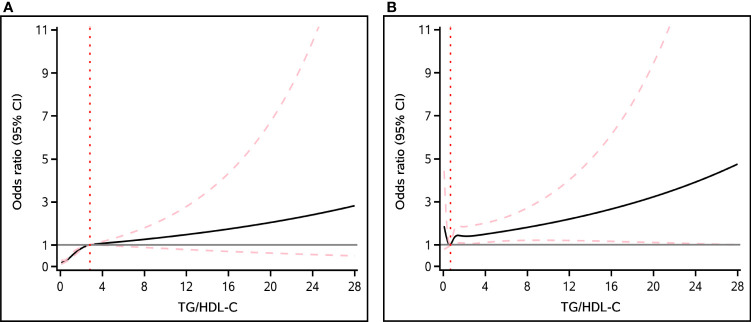
The nonlinear association between TG/HDL-C ratio and the risk of NAFLD **(A)** and liver fibrosis **(B)**. Solid black line represents the smooth curve fit between variables. The pink dashed lines represent the 95% of confidence interval from the fit.

By smooth curve-fitting analysis, we further investigated the potential nonlinear relationship between TG/HDL-C ratio and NAFLD and liver fibrosis. According to **Figure 2**, our findings indicated a nonlinear positive correlation between the TG/HDL-C ratio and NAFLD, while such a relationship does not exist in liver fibrosis.

### 3.5 Subgroup analysis

**Figure 3:**
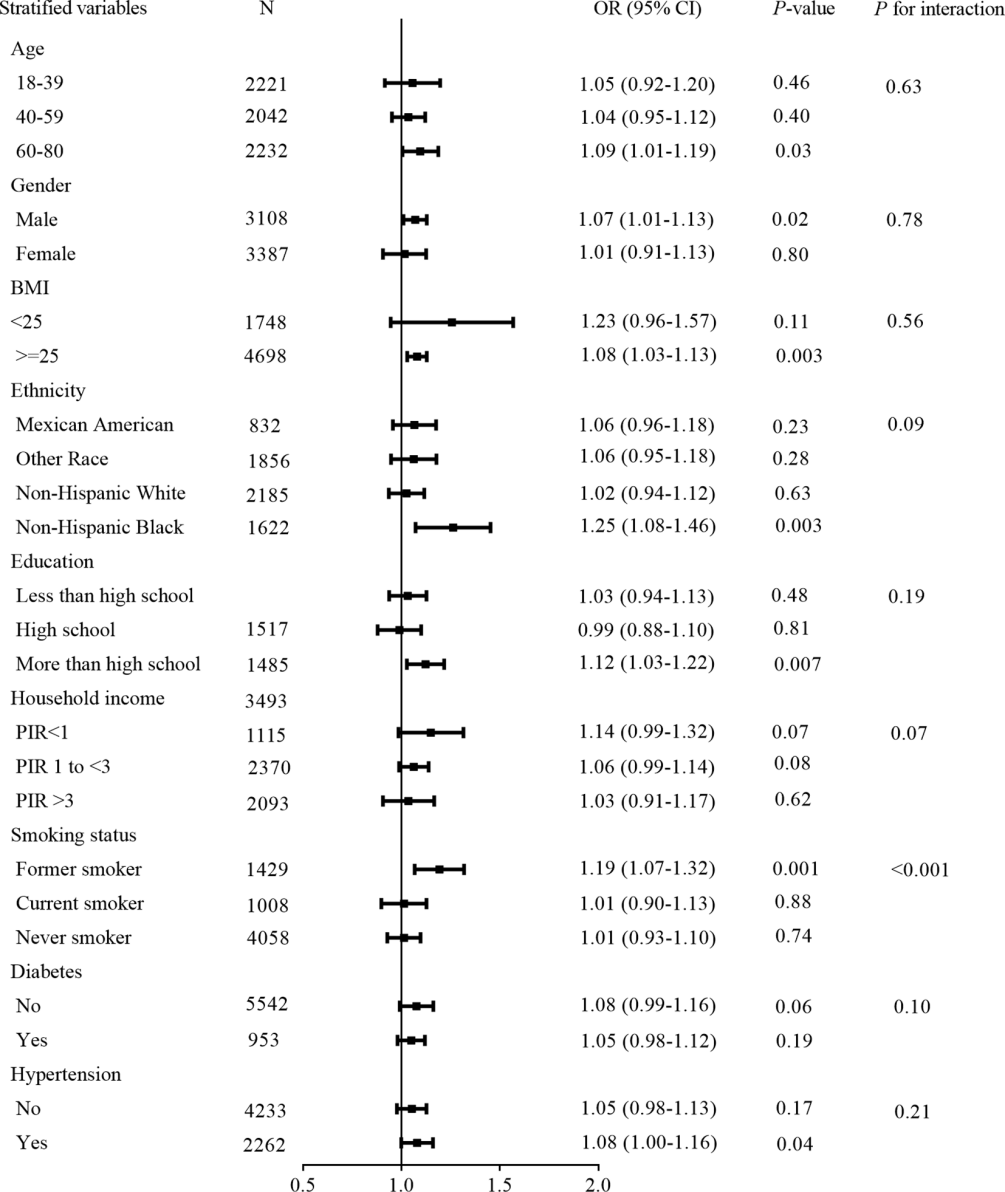
Stratified analysis for NAFLD.

**Figure 4:**
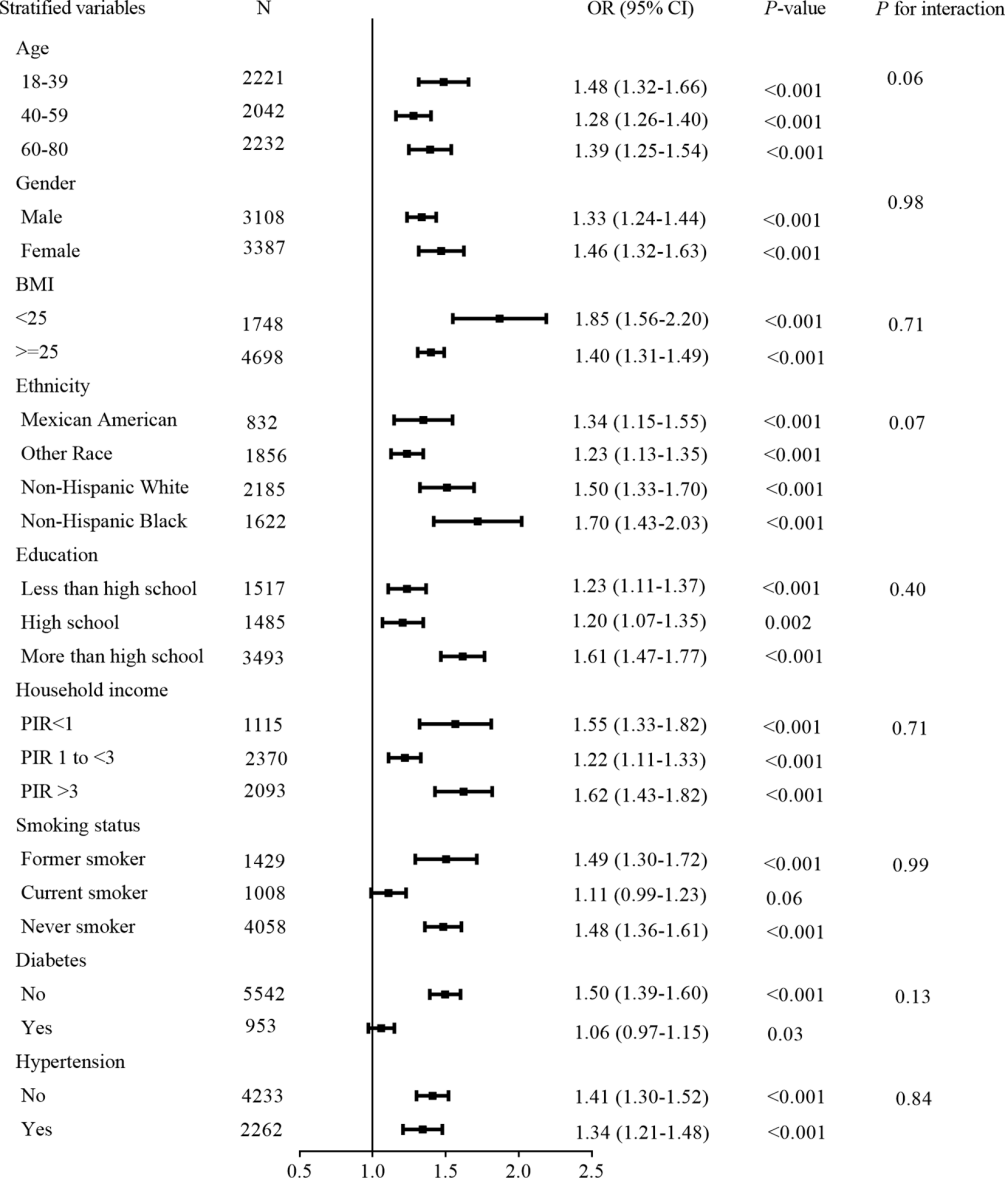
Stratified analysis for liver fibrosis.

Stratified analyses were performed based on age, sex, BMI, ethnicity, smoking status, educational attainment, household income, hypertension, and diabetes. The results of stratified analysis of NAFLD and liver fibrosis are shown in **Figures 3** and **4**.The positive correlation between TG/HDL-C ratio and NAFLD exhibited a broad consistency across populations. In addition, we found an interaction between smoking status and TG/HDL-C ratio in relation to the prevalence of liver fibrosis (*P* for interaction < 0.001).

## 4 Discussion

NAFLD is recently prevalent worldwide, and timely and effective assessment of NAFLD risk is critical for patients, with efforts to stop disease progression to advanced fibrosis or hepatocellular carcinoma (37). Currently, liver biopsy remains the established standard to assess the extent of liver steatosis and fibrosis is; however, being a harmful and invasive procedure, liver puncture should therefore be cautiously applied in clinical practice (38). LUTE is widely used for evaluating the extent of liver fibrosis and steatosis; its main advantage is that it is noninvasive, and the results are comparable to those of a liver biopsy (39, 40). Despite limited evidence on the relationship between TG/HDL-C ratio and NAFLD (29, 30, 41), it remains unclear how this ratio is linked to NAFLD and liver fibrosis in American adults. The current study examined this association in American adults, with the results demonstrating a positive correlation between TG/HDL-C ratios and NAFLD in all models. Grouping the ratios into quartiles revealed a consistent correlation that strengthened with increasing TG/HDL-C. However, although the TG/HDL-C ratios were positively correlated with liver fibrosis in all models, this positive correlation was not statistically significant in Model 3. Our findings could assist healthcare professionals in identifying high-risk patients for NAFLD and liver fibrosis, thereby enabling more informed decisions regarding patient management.

Recently, increasing evidence has suggested that TG/HDL-C ratios were linked to more unfavorable metabolic diseases, such as atherosclerosis, obesity, diabetes, and dyslipidemia (41). Our results were consistent with previous results and also showed that for participants with higher TG/HDL-C ratios, LDL-C, AST, ALT, TC, GGT, Alb, TG, CRP, blood pressure, BMI, and ferritin levels were significantly higher compared with those who presented lower TG/HDL-C ratios.

However, the mechanism underlying the link between TG/HDL-C ratio and NAFLD and liver fibrosis have not been fully elucidated. IR is an important feature of NAFLD and may, therefore, be a potential mediator. IR and lipid disorders are the starting links and the core of NAFLD. When the effector organs of insulin action (e.g., liver, skeletal muscle, adipose tissue) become less sensitive to insulin, there is a compensatory increase in insulin, eventually leading to hyperinsulinemia. Compensatory elevation of insulin can promote hepatic synthesis of TC, which also elevates LDL-C levels and leads to inactivation of lipoprotein lipase, which reduces clearance of TG. Consequently, large amounts of TG accumulate in the liver (42). Additionally, when the concentration of TG is elevated in the bloodstream, the “debris” and free fatty acids of TG celiacs transported to the liver through the bloodstream will increase, which exceeds the liver’s ability to utilize these fatty acids, ultimately triggering the development of NAFLD (43–46). Moreover, IR leads to decreased fat metabolism and increased catabolism, and the liver takes up large amounts of free fatty acids. However, the β-oxidation of fatty acids is inhibited by hyperinsulinemia, and a large amount of free fatty acids accumulates in the liver, exacerbating hepatocellular steatosis (47). As NAFLD progresses, oxidative stress and excessive lipid deposition aggravate the impairment of hepatocyte mitochondrial function, which further attenuates the metabolism of lipid pairs, thereby leading to further increases in the levels of TG, TC, and other markers (47). IR induces the secretion of larger and TG-overconcentrated very low-density lipoprotein particles while decreasing HDL-C concentration (48, 49). Therefore, IR contributes to an increase in TG/HDL-C ratio.

Recently, a strong association between IR and leptin secretion has been reported (50). Leptin is a protein-like hormone secreted by adipose tissue, and the degree of hepatic steatosis is positively correlated to its expression level (50). Persistent hyperleptinemia has therefore been linked to steatosis, liver fibrosis, and hepatocellular cancer in several observational clinical investigations, indicating that hyperleptinemia is a reliable indicator of the onset or development of NAFLD (51–53). It is currently believed that leptin promotes the development of NAFLD by centrally suppressing dietary intake and increasing sympathetic nerve activity, metabolic rates, and gluconeogenesis (54, 55).

This study has significant clinical value, as it reliably assesses hepatic steatosis and fibrosis in American adults with an adequate and representative sample size. However, certain limitations were also noted. Firstly, being a cross-sectional study, the causal association between TG/HDL-C ratio and NAFLD and liver fibrosis could not be elucidated. A longitudinal study in the future is necessary to better investigate the potential causal relationships. Secondly, the findings may not be applicable to individuals aged < 18 years since all included participants were over 18 years old. Therefore, further studies are required for investigating the association between TG/HDL-C ratios and NAFLD and liver fibrosis in this population. Thirdly, despite the fact that 6,495 participants have been included in this research, there is still a risk of insufficient sample size for a specific group, and therefore it is necessary for future studies to include more participants to demonstrate our results. Fourthly, the inherent limitations of the NHANES database resulted in several confounders that may have had an effect on the results that were not adjusted for. Finally, there are several potential influences on the occurrence and development of NAFLD and liver fibrosis. Despite including as many covariates as possible in this study and making adjustments to the models, the possibility that unaccounted confounders could have led to biased results cannot be excluded. Therefore, additional prospective studies would be required to validate the findings of this investigation and address the above limitations.

## 5 Conclusions

In the American adult population, the TG/HDL-C ratio was nonlinearly and positively associated with the prevalence of NAFLD; however, this relationship was not present in liver fibrosis.

## Data availability statement

The raw data supporting the conclusions of this article will be made available by the authors, without undue reservation.

## Ethics statement

National Center for Health Statistics Ethics Review Committee gave approval for the NHANES survey protocol, with written informed consent also obtained from all participants. Since the NHANES database is publicly available, this study was exempted from ethical review. The studies were conducted in accordance with the local legislation and institutional requirements. Written informed consent for participation was not required from the participants or the participants’ legal guardians/next of kin in accordance with the national legislation and institutional requirements.

## Author contributions

JW: Writing – review & editing, Writing – original draft, Validation, Funding acquisition, Formal analysis, Data curation. HLi: Writing – original draft, Investigation, Data curation, Conceptualization. XW: Writing – original draft, Visualization, Validation, Software, Resources, Project administration, Conceptualization. RS: Writing – original draft, Supervision, Software, Methodology, Investigation, Data curation. JH: Writing – original draft, Validation, Software, Project administration, Investigation, Formal analysis, Conceptualization. XZ: Writing – review & editing, Visualization, Validation, Supervision, Resources, Methodology, Data curation. HAL: Writing – review & editing, Validation, Project administration, Methodology, Formal analysis, Data curation. PY: Writing – review & editing, Software, Methodology, Investigation, Data curation, Conceptualization. HWL: Writing – original draft, Project administration, Methodology, Data curation. YC: Writing – original draft, Software, Investigation, Conceptualization. XC: Writing – original draft, Data curation. SC: Writing – original draft, Supervision, Investigation, Funding acquisition, Formal analysis. DW: Writing – review & editing, Writing – original draft, Conceptualization.
